# How a New Understanding of Drug or Drug Class Pharmacology Often Drives Drug Development: A Conversation with Steven E. Nissen, MD

**DOI:** 10.14797/mdcvj.1181

**Published:** 2022-12-06

**Authors:** Steven E. Nissen, James B. Young

**Affiliations:** 1 Chief Academic Officer of the Cleveland Clinic Heart, Vascular & Thoracic Institute, Cleveland, Ohio, US; 2 Executive Director of Academic Affairs, Cleveland Clinic, Cleveland, Ohio, US

**Keywords:** drug development, US Food and Drug Administration (FDA) drug approval, clinical trials

## Abstract

This video interview features Steven Nissen, MD, telling his 2007 story of revealing that the number-1 diabetes drug, rosiglitazone, led to more cardiovascular events than placebo or a variety of competitors. The discovery resulted in new requirements for clinical trials to rule out harm. It focuses on how a new understanding of drug or drug class pharmacology often drives drug development, and includes a discussion of the approval process, which may bring a different result than was originally intended. View the video at https://youtu.be/aFtCfZyd54E.

## Introduction

Steven E. Nissen is a cardiologist, researcher, and patient advocate who joined the Cleveland Clinic in 1992. He was vice-chairman of the Department of Cardiology (1993-2002), section head of Clinical Cardiology (1992-2000), and director of the Coronary Intensive Care Unit (1992-1997). He still rounds regularly in that unit. He has served as medical director of the Cleveland Clinic Cardiovascular Coordinating Center directing multicenter clinical trials. A native of Southern California, Nissen attended undergraduate and medical school at the University of Michigan, completed internal medicine training at the University of California, Davis, in Sacramento, and a cardiology fellowship at the University of Kentucky. He was an early proponent of intravascular ultrasound to document and quantify coronary artery disease (CAD) progression and regression. His studies led to a seminal observation that CAD could be diminished with intensive lipid modulating therapies. He has led numerous clinical trials, including those linking the COX-2 (cyclooxygenase-2) inhibitor Vioxx (rofecoxib) to an increased risk of heart attacks and strokes in 2001. In 2005 Nissen reanalyzed data regarding Pargluva (muraglitazar), an experimental type 2 diabetes drug at the time, finding significant concerns. In 2007 a meta-analysis by Nissen found that the diabetes drug rosiglitazone (Avandia) also carried high cardiovascular risk. Nissen subsequently championed large-scale CVD (cardiovascular disease) morbidity/mortality trials for all new DM (diabetes mellitus) drugs which historically had been evaluated primarily by glucose modulating studies. This led to the more recent clinical trials that have dramatically changed the paradigm and guidelines for treating CVD, and CHF (congestive heart failure) in particular, with SGLT2 (sodium-glucose cotransporter-2) inhibitors of note. Dr. Nissen served on the US Food and Drug Administration (FDA) Cardio-Renal Advisory Panel (CRAP) from 2000-2005 and was chairman for his final year. He was president of the American College of Cardiology from 2006-2007 and also in 2007 he was named one of the 100 Most Influential People in the world by *Time* magazine. Dr. Nissen’s career has remained prolific, and he is a much-loved clinician, educator, and investigator at the Cleveland Clinic. He is currently the Chief Academic Officer of the Sydell and Arnold Miller Family Heart, Vascular, and Thoracic Institute, and he holds the Dickey Chair in Cardiovascular Medicine. He is a dear friend, colleague, and collaborator of many.

## Interview

**Figure 1:**
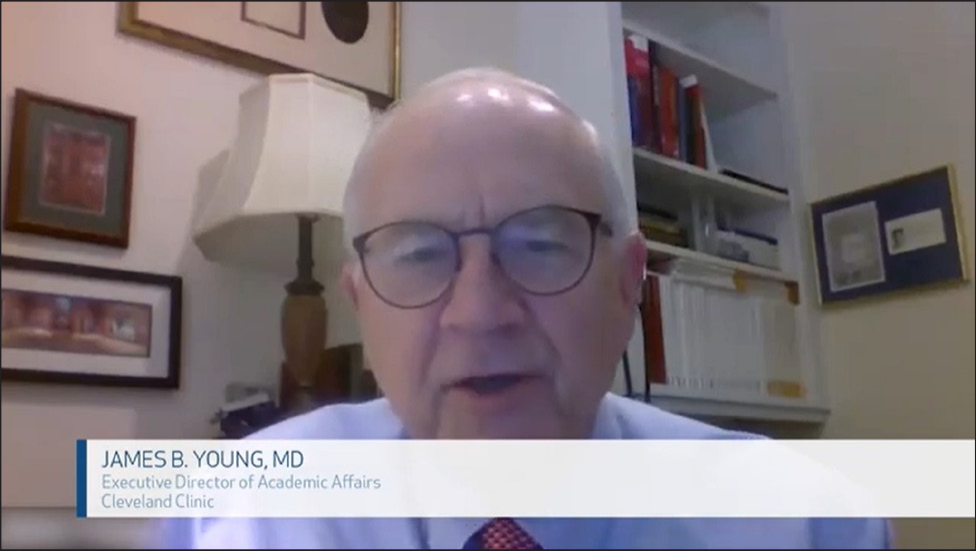
James Young, MD, conducted this interview, which focuses on the paradigm shift toward conducting large-scale cardiovascular end point trials for drugs that are not primarily developed for that purpose. View the video at https://youtu.be/aFtCfZyd54E.

James Young (Figure 1): I want to welcome all of the people who are joining us for what’s going to be, I know, a spirited conversation with Dr. Steve Nissen. We’re very happy to have you here, Steve.

What I’m going to ask you—since the *Journal* is focusing its upcoming issue on cardiovascular pharmacology and new indications for drugs, particularly newer agents for prevention and treatment of cardiovascular disease in general, and heart failure specifically—are your thoughts on drug development and the FDA process. Can you tell me how you got excited and interested in this subject, and how you forced a paradigm shift with the FDA requiring new types of trials?

**Figure 2:**
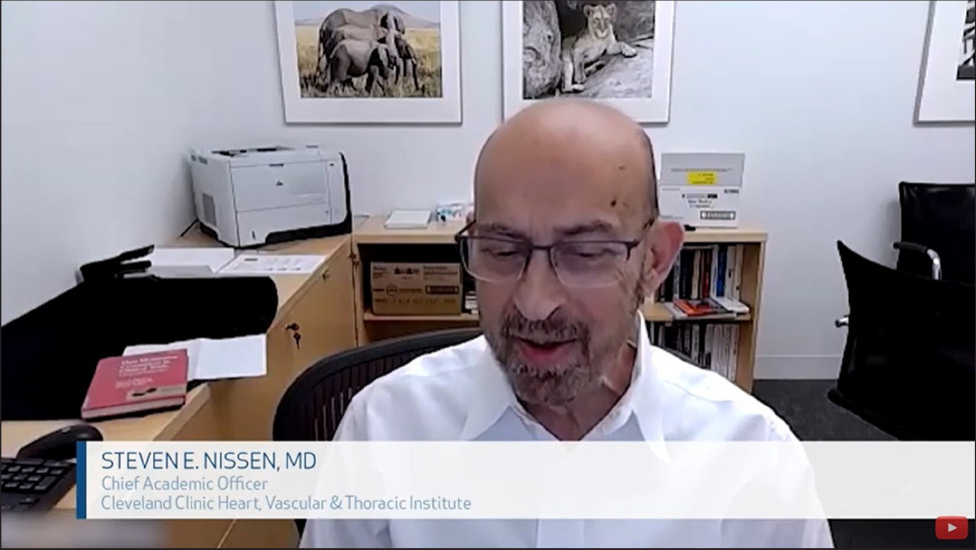
Steven Nissen, MD, shares his story of revealing in 2007 that the number-1 diabetes drug, rosiglitazone, led to more cardiovascular events than placebo or a variety of competitors. The discovery resulted in new requirements for clinical trials to rule out harm. View the video at https://youtu.be/aFtCfZyd54E.

Steven Nissen (Figure 2): Well, first of all, thank you for that extraordinarily kind introduction. You did leave off one thing. I did chair the Department of Cardiovascular Medicine for 13 years. It was a very challenging job and I’m happy that I don’t have to worry about it anymore.

What happened was I became interested in the TZDs—the thiazolidinediones—and actually I did an intravascular ultrasound trial with one of them, pioglitazone. Several trials were done with rosiglitazone, known by their brand name of Avandia. And you know, in perusing those trials because of my interest in the class, I noticed that although the trials were not cardiovascular outcome trials, the cardiovascular events trended in the wrong direction: There were more events with rosiglitazone than with placebo.

And so I was interested in taking a look at this issue because I had been aware, because of the muraglutazar story, that this class of drugs could increase cardiovascular events. And my dear colleague and statistician Kathy Wolski and I perused the internet and eventually found a treasure.

There was a website where the maker of rosiglitazone had posted all of their clinical trial data and this happened for a very specific reason. The company GlaxoSmithKline had been sued by Eliot Spitzer, the attorney general of New York, because they had failed to publish negative trials on one of their antidepressant drugs. Part of the settlement was that they had to disclose the results of all their trials. I found more than 40 clinical trials on this website, about two-thirds of which had never been published.

We downloaded all the information—it was study level—and Ms. Wolski and I analyzed the data. In almost every study, there were more cardiovascular events with rosiglitazone than with a whole variety of competitors. We quickly wrote a manuscript—we thought this was serious—because at the time rosiglitazone was the number-1-selling diabetes drug in the world. It was hugely popular and it was popular because it was purported to have cardiovascular benefits. We sent that manuscript to the *New England Journal of Medicine*, and the editors at the time understood its importance.^1^ They sent our meta-analysis, which had shown a very substantial increase in the risk of myocardial infarction that was a significant (*P* = .06) increase in cardiovascular death, out for a large number of reviews.

We rapidly, over just a matter of days, went through several cycles of review and they finally, on May 21st 2007, published the manuscript. The result created a storm, an enormous storm. It was the lead story on the front page of the *Wall Street Journal*. I couldn’t keep up with the requests for interviews.

I made a mistake in not telling the FDA this was coming, which was historically a problem because when they get blindsided they often react defensively. And this was not the cardiorenal division, this was the endocrine metabolism division. Nonetheless, this was published and it was controversial. I presented in a plenary at the ADA (American Diabetes Association) meeting, and a few months after that article appeared, the FDA did their own analysis, found the same thing, and we later learned that the company had also done a meta-analysis and got the same result.

So the FDA in 2008 said, “What should we do?” and they called an advisory committee meeting and had three external experts give talks: me, Rury Holman, who had done the UKPDS study (United Kingdom Diabetes Study)^2^—a very famous diabetologist—and Rob Califf, now FDA Commissioner.

And I got up and made a presentation. I set up the slide set where I said, “We cannot go along with the idea that simply because a drug lowers a biomarker, glucose, that it should be approved for patients with diabetes.” We needed to understand the positive and negative effects of these drugs in cardiovascular outcome trials given what we had seen with rosiglitazone.

I made what I thought was a strong presentation, and I suggested a two-step process where trials would first have to rule out a fairly high level of harm, a relative risk of 1.8. And then, subsequently, they would have to rule out a relative risk of 1.3 in either a second trial or with an ongoing trial.

Rury Holman got up—he hadn’t seen my slides previously—and he focused on the fact that he agreed with the recommendation. And then Rob Califf got up and said, “I agree with Steve Nissen’s recommendation.” And to my utter shock, this committee of diabetologists voted to recommend to the FDA that the process I had outlined should be followed.

Now there’s something important in that slide set: the last couple of slides said “Look, if we do studies to rule out harm, there’s a very good chance that one or more of them will actually show benefit.” These are large outcome trials, you know, 4, 5, 6,000—even more—patients, and I was well aware that there might be new drugs. By the way, this was widely criticized in the diabetes community. They said, “We will have no new diabetes drugs because Steve Nissen convinced the FDA to require outcome trials,” which, of course, was completely wrong.

The first trials showed essentially no benefits and no risks, and they were the DPP-4 (Dipeptidyl peptidase 4) inhibitors—I believe a worthless class of drugs as they don’t do anything to improve outcomes and barely allow for blood sugar control. I don’t know why they’re continuing to be used. But then something else happened.

The first of the SGLT2 inhibitor trials to report—the EMPA-REG trial with empagliflozin—showed a fairly compelling cardiovascular benefit, including a reduction in death.^3^ And next the GLP-1 (glucagon-like peptide 1) agonists were studied in clinical trials. The first was with liraglutide, and I served on the executive committee for that study, and it showed benefit. And what happened was—because of the requirement to do outcome trials—we transitioned from the glucose-centric era, where all you had to do was show you lowered blood sugar, to an era where now you can’t do a trial of a new drug for diabetes without comparing it to one of the other drugs shown to be beneficial—either an SGLT2 inhibitor or a GLP-1 agonist.

So the entire landscape of diabetes drugs changed. I will tell you I’m probably most proud of having done that than anything else I did. I took a terrible beating over this. I mean, statisticians, diabetologists…they all just…I was assaulted over this. But now I think, looking back, people understand that it was the right thing to do.

James Young: So Steve, I have to say that I was a bystander watching all of this. As you recall, I spent a decade doing an odd, a very odd job for a cardiologist: I was the chair of the Cleveland Clinic’s Diabetes and Metabolism Institute at the time. And it was groundbreaking to watch this. You were very brave, indeed. You weathered the storm. But for me, the seminal thing that you accomplished was application of large-scale cardiovascular end point trials to other drugs that were not primarily being developed for that particular purpose. And you have changed the paradigm dramatically, and so I really applaud you for doing that.

We’re ending the time we have and I’ll give you one last moment for a final comment to summarize things, but I think you did a great job telling the story. People need to listen to this story and apply it to other areas. There is no question about that.

Steve Nissen: I’ll just make one final comment: That’s exactly what we’re now doing. We are pivoting to outcome trials for obesity drugs. And, you know, this has now become the standard approach, and so I’m very happy to see that, and I think we will see some remarkable outcomes in the next few years.

James Young: Well, Steve, I want to thank you very much. On behalf of the editorial board for the *Methodist DeBakey Cardiovascular Journal*, this has been a great conversation and you’ve made some extraordinarily important points. Thank you so much.

Steven Nissen: Thank you so much, too.
